# Magnetic poly(2-hydroxyethyl methacrylate) microspheres for affinity purification of monospecific anti-p46 kDa/Myo1C antibodies for early diagnosis of multiple sclerosis patients

**DOI:** 10.1042/BSR20160526

**Published:** 2017-04-28

**Authors:** Daniel Horák, Helena Hlídková, Yurii Kit, Volodymyr Antonyuk, Severyn Myronovsky, Rostyslav Stoika

**Affiliations:** 1 Institute of Macromolecular Chemistry, AS CR, Heyrovsky Sq. 2, 162 06 Prague 6, Czech Republic; 2 Institute of Cell Biology, NAS of Ukraine, Drahomanov Str. 14/16, Lviv 79005, Ukraine

**Keywords:** affinity purification, anti-p46 kDa/Myo1C, magnetic microspheres, multiple sclerosis

## Abstract

The aim of the present study is to develop new magnetic polymer microspheres with functional groups available for easy protein and antibody binding. Monodisperse macroporous poly(2-hydroxyethyl methacrylate) (PHEMA-COOH) microspheres ~4 µm in diameter and containing ∼1 mmol COOH/g were synthesized by multistep swelling polymerization of 2-hydroxyethyl methacrylate (HEMA), ethylene dimethacrylate (EDMA), and 2-[(methoxycarbonyl)methoxy]ethyl methacrylate (MCMEMA), which was followed by MCMEMA hydrolysis. The microspheres were rendered magnetic by precipitation of iron oxide inside the pores, which made them easily separable in a magnetic field. Properties of the resulting magnetic poly(2-hydroxyethyl methacrylate) (mgt.PHEMA) particles with COOH functionality were examined by scanning and transmission electron microscopy (SEM and TEM), static volumetric adsorption of helium and nitrogen, mercury porosimetry, Fourier transform infrared (FTIR) and atomic absorption spectroscopy (AAS), and elemental analysis. Mgt.PHEMA microspheres were coupled with p46/Myo1C protein purified from blood serum of multiple sclerosis (MS) patients, which enabled easy isolation of monospecific anti-p46/Myo1C immunoglobulin G (IgG) antibodies from crude antibody preparations of mouse blood serum. High efficiency of this approach was confirmed by SDS/PAGE, Western blot, and dot blot analyses. The newly developed mgt.PHEMA microspheres conjugated with a potential disease biomarker, p46/Myo1C protein, are thus a promising tool for affinity purification of antibodies, which can improve diagnosis and treatment of MS patients.

## Introduction

Rapid and effective magnetic separation and manipulation of various biological entities, including proteins (antibodies and enzymes) and cells, as well as drug targeting and delivery, require involvement of biocompatible magnetic carriers [1]. Magnetic separation offers higher throughput and specificity than other isolation methods, such as centrifugation or filtration, because magnetic particles can be easily removed from complex mixtures using a magnetic field [2]. The magnetic particles are preferably based on naturally occurring non-toxic iron oxides, such as maghemite (γ-Fe_2_O_3_) and magnetite (Fe_3_O_4_) [3]. To avoid detrimental effects, such as Fe ion leaching, aggregation in aqueous media, insufficient compatibility with living tissues, and absence of functional groups available for attachment of a target biomolecule, proper coating of the magnetic particles with polymers is needed [4]. This coating can be achieved by simple adsorption [5], graft polymerization [6,7] and co-polymerization from the particle surface [8] or iron oxide encapsulation by solvent evaporation [9], microemulsion [10], and miniemulsion polymerization [11]. Some of these techniques offer production of spherical particles, which are preferred to other shapes, such as cylinders or cubes, as they provide higher surface-to-volume ratio for separation of analytes and reagents. This enables quick antibody–antigen conjugation and reduces both the reaction volume and incubation time [12–14].

Magnetic polymer microspheres are commonly synthesized from a variety of materials, such as polystyrene [15], poly(methyl methacrylate) [16], poly(glycidyl methacrylate) [17], and conveniently functionalized with COOH, NH_2_, SH, or other groups to easily immobilize a biomolecule, e.g. antibody [18]. The advantage of poly(2-hydroxyethyl methacrylate) (PHEMA) as a matrix of magnetic microspheres is its biocompatibility documented by a long biomedical history of use in artificial embolization [19,20], surgery, cell affinity chromatography [21], and drug release [22]. Compared with polystyrene, PHEMA has the advantage that aromatic benzene rings are absent, minimizing autofluorescence, which could disturb analysis. It is also convenient that PHEMA microspheres can have a porous structure, which enables introduction of magnetic compounds.

Magnetic polymer particles are preferably used for purification of tiny quantities of proteins in blood serum [23–25]. Enrichment of specific proteins in biological samples helps identify disease specific biomarkers at early stages [24,25]. Our previous studies were focused on identification of protein markers in the blood serum of patients suffering from multiple sclerosis (MS), rheumatoid arthritis (RA), and systemic lupus erythematosus (SLE) [26,27]. The original precipitation/extraction method and MALDI TOF/TOF mass spectrometry was therefore developed for isolation of the human unconventional myosin IC isoform b (p46/Myo1C) fragment (*M*_r_ ∼46 kDa) as a potential marker of the listed autoimmune diseases [26]. High p46/Myo1C levels were found in the blood serum of MS and RA patients and low amounts were found in SLE patients, while this protein was not detected in the blood serum of healthy subjects.

To develop new immunodiagnostic approaches for rapid quantification of p46/Myo1C in blood serum, it is important to have monospecific antibodies directed against this protein. However, target antigens are often contaminated by other proteins and a lot of effort, not always successful, is required to separate them. To solve these problems, magnetic microspheres containing specific protein antigens are needed to allow easy isolation of the monospecific antibodies [27].

The aim of the present study is to synthetize monodisperse magnetic poly(2-hydroxyethyl methacrylate) (mgt.PHEMA) microspheres with COOH functionality. The p46/Myo1C protein from the blood serum of MS patients is then conjugated with the particles, and affinity isolation of monospecific anti-p46 kDa/Myo1C antibodies from crude antibody preparation is performed. This purification approach seems to be very promising for early diagnosis of MS.

## Materials and methods

### Materials

Monomers, including 2-hydroxyethyl methacrylate (HEMA; Röhm; Darmstadt, Germany) and ethylene dimethacrylate (EDMA; Ugilor, France), were distilled under vacuum. 2-[(Methoxycarbonyl)methoxy]ethyl methacrylate (MCMEMA) was synthesized from ethylene glycol, chloroacetic acid, methanol, and methacrylic anhydride according to published procedures [28]. 2,2′-Azobis(2,3,3-trimethylbutanonitrile) (ABTB) was prepared from 3,3-dimethylbutan-2-one, hydrazine sulfate, sodium cyanide, bromine, and recrystallized from ether [29]. Cyclohexyl acetate (CyAc) was obtained by reaction of cyclohexanol and acetic anhydride. Methocel 90 HG [(hydroxypropyl)methylcellulose], dibutyl phthalate (DBP), sodium dodecyl sulfate (SDS), and trichloroacetic acid (TCA) were from Fluka (Dorset, U.K.). FeCl_2_·4H_2_O, blood serum albumin (BSA), 3,3′-diaminobenzidine (DAB), *N,N*′-diisopropylcarbodiimide (DIC), Ponceau S, and buffers for biological experiments were from Sigma–Aldrich (St. Louis, U.S.A.). Other chemicals were supplied by Lach-Ner (Neratovice, Czech Republic). Ultrapure Q-water ultrafiltered on a Milli-Q Gradient A10 system (Millipore; Molsheim, France) was used in all experiments.

### Magnetic PHEMA microspheres

Magnetic PHEMA microspheres were prepared by modifications of earlier described procedures [30–32]. To obtain monodisperse macroporous PHEMA microspheres, multistep swelling polymerization of HEMA (40 wt.%), MCMEMA (20 wt.%), and EDMA (40 wt.%) using polystyrene seeds was run in the presence of inert solvents (porogens), such as CyAc and DBP. Polystyrene latex was obtained by the emulsifier-free emulsion polymerization and dispersed in an emulsion of DBP in aqueous SDS solution. The latex (1.2 g of polystyrene) was stirred (30 rpm) with an emulsion of ABTB (0.12 g), HEMA (4.8 g), MCMEMA (2.4 g), and EDMA (4.8 g) in aqueous 0.1% SDS (30 ml) for 16 h. CyAc (16.8 g) was sonicated (4710 Series Cole-Parmer Ultrasonic Homogenizer, Chicago, U.S.A.; 10 W) in 0.1% SDS (30 ml) for 3 min and then added to the above suspension. The mixture was stirred (300 rpm) for 1 h, 2 wt.% aqueous Methocel 90 HG solution (12 ml) was added under CO_2_ atmosphere, and the mixture was polymerized at 70°C for 16 h with agitation (400 rpm). The resulting macroporous MCMEMA-containing PHEMA microspheres were washed five times with 0.01 wt.% Tween 20 and ethanol. To introduce COOH groups, the particles were hydrolyzed with 0.4 M aqueous NaOH (120 ml) at RT for 48 h with stirring (50 rpm) and at 70°C for 16 h. The PHEMA-COOH microspheres were repeatedly washed with water, acetone, 20% ethanol, and water.

To render the PHEMA-COOH microspheres with magnetic properties, magnetite (Fe_3_O_4_) and/or maghemite (γ-Fe_2_O_3_) was precipitated within the pores of the polymer matrix. Briefly, an FeCl_2_ solution was imbibed several times into HCl-acidified macroporous PHEMA-COOH microspheres under Ar atmosphere. The particles were separated and redispersed in NH_4_OH solution under Fe_3_O_4_ formation. This procedure was followed by rinsing with water, slow oxidation of Fe_3_O_4_ to γ-Fe_2_O_3_ in air with shaking, and multiple washes with water until iron oxide colloid formation occurred within the microspheres, which were termed mgt.PHEMA.

### Characterization of particles

Particle size and morphology were investigated using a Quanta 200 FEG SEM microscope (FEI; Brno, Czech Republic) at accelerating voltage of 30 kV. SEM micrographs were evaluated to assess uniformity of the microspheres by determining polydispersity index, PDI = *D*_w_/*D*_n_, where $D_{w}=\Sigma n_{i}D_{i}^{4}/\Sigma n_{i}D_{i}^{3}$, *D*_n_ = Σ *n*_i_*D*_i_/Σ *n*_i_; *D*_n_ and *D*_w_ are the number- and weight-average particle diameters of at least 500 particles (Atlas software; Tescan; Brno, Czech Republic). To monitor the inner structure of the magnetic microspheres, they were fixed in London Resin White and cut with a LKB III ultramicrotome (Leica Biosystems; Wetzlar, Germany). The ultrathin sections were observed on carbon-coated copper grids by a Tecnai G2 Spirit Twin 12 transmission electron microscope (TEM; FEI) at accelerating voltage of 120 kV. Volume of particle pores (*V*_p_ < 200 nm) was determined by single-point static volumetric adsorption of helium (at relative pressure, *p*/*p*_0_ = 0.99) on a Gemini VII 2390 analyzer (Micromeritics; Norcross, GA, U.S.A.). Specific surface area (*S*_BET_) was measured by multiple-point nitrogen adsorption on the same instrument, and diameter of the pores (*d*) was calculated as (*d* = 4 × *V*_p_/*S*_BET_). Porosity (*ε*) was determined as *ε* = (*V*_p_ × 100)/(*V*_p_ + 1/ρ), where ρ is the pycnometrically determined PHEMA density (1.3 g/ml) [33]. Pore structure of dry PHEMA-COOH microspheres was evaluated on Pascal 140 and 440 mercury porosimeters (Thermo Finnigan; Rodano, Italy) at 0–400 kPa and 1–400 MPa, enabling detection of meso- and macropores [34]. Cumulative pore volume (*V*_c_), pore diameter (*d* < 200 nm), and porosity were calculated by Washburn’s equation for capillary flow in cylindrical pores [35]. Water (WR) and cyclohexane regain (CXR) of equilibrium-swollen PHEMA-COOH microspheres corresponding to total pore volume (*V*_t_) were determined by suction and centrifugation. Porosity was calculated as described above [36,37]. Carbon, nitrogen, and iron content in the microspheres were quantified by a Perkin-Elmer 2400 CHN elemental analyzer (Waltham, U.S.A.) and a Perkin-Elmer 3110 atomic absorption spectrometer (AAS). Fourier transform infrared (FTIR) spectra were measured on the diamond crystal with a 45° angle of incidence using a Perkin-Elmer Paragon 1000PC spectrometer with a Specac MKII Golden Gate single attenuated total reflection (ATR) system. Each sample was scanned 64 times at 4,400–450 cm^−1^ (resolution 4 cm^−1^).

### Animal immunization

White laboratory mice [38] were maintained in pathogen-free animal facilities with enough water and food. Animals were immunized with p46/Myo1C protein (100 µg) purified from the blood serum of MS patients [26] for 8–12 weeks; the immunization was repeated after 2 and 4 weeks [38].

### Preparation of p46/Myo1C-mgt.PHEMA microspheres

The p46/Myo1C protein was isolated from the blood serum of MS patients, as described previously [26], and purified by a Series 200 HPLC gel filtration (Perkin-Elmer; U.S.A.) on a Bio-Sil SEC 250 column (Bio-Rad; Marnes-la-Coquette, France) in phosphate buffer (150 mM NaCl, 10 mM Na_2_HPO_4_, 5 mM NaH_2_PO_4_; pH 6.8) at a flow rate of 1 ml/min. The column was calibrated by molecular mass (*M*_r_) standards in the same buffer. Protein fractions were concentrated to 1 mg/ml by an Amicon Ultra-0.5 centrifuge filter (Millipore).

Magnetically separated mgt.PHEMA microspheres (0.18 ml) were washed with 0.1 M carbonate buffer (pH 9.0) and 0.1 M acetate buffer (pH 5.4) three times each. A solution of DIC (12.5 µl) in acetate buffer/DMSO (both 0.125 ml) was immediately added to the particles, and the suspension was incubated for 160 min. The microspheres were washed twice with acetate buffer/DMSO mixture (85:15 v/v) and 0.2 M borate buffer (pH 8.5). A solution of p46/Myo1C protein (0.6 mg) in borate buffer (0.3 ml) was added to the particles, and the mixture was incubated for 18 h. Determination of the residual protein concentration in the supernatant revealed that 0.45 mg of p46/Myo1C was bound to the particles, corresponding to 7 mg of protein per ml of the particle suspension.

### Antibody purification and characterization

Crude antibody preparation was obtained from the blood serum (500 µl) of immunized mice by precipitation with 33% (NH_4_)_2_SO_4_. To obtain monospecific anti-antibody, a crude antibody preparation (150 µl; 3.6 mg/ml) was incubated with p46/Myo1C-mgt.PHEMA (300 µl) at RT for 2 h with stirring. The microspheres were washed with Tris-buffered saline (TBS), 0.01% Tween 20, TBS (twice), and the bound anti-p46/Myo1C antibodies were eluted with 0.1 M glycine-HCl buffer (100 µl). Eluted proteins were then dialyzed against PBS, and their concentration was determined from UV spectra measured on a NanoDrop ND1000 spectrophotometer (Wilmington, DE, U.S.A.) at 280 nm. This was followed by SDS/10% PAGE (sodium dodecyl sulfate-polyacrylamide gel electrophoresis), protein blotting on a nitrocellulose membrane (Thermo Fischer Scientific; Waltham, MA, U.S.A.), and incubation with IgG fraction obtained by precipitation with 33% (NH_4_)_2_SO_4_ from mouse anti-p46/Myo1C serum.

Dot blot analysis was carried out with the antigen titer in the range of 2–0.065 µg on spot. The crude and affinity isolated p46/Myo1C antibodies were diluted with PBS (1:250 v/v). Western blot and dot blot analyses were performed according to the following scheme: incubation with primary antibodies in PBS for 1 h, washing with PBS/0.1 wt.% Tween 20 (3 × 5 min), incubation with peroxidase-labeled secondary antibodies for 1 h, washing with PBS/0.1 wt.% Tween 20 (3 × 5 min), staining with DAB for 10 min, and addition of H_2_O_2_/PBS solution (4/1 ml/ml).

### Bioethics

Blood serum of MS patients diagnosed according to the McDonald diagnostic criteria for MS, provided by Tetyana Nehrych and Nazar Negrych from Danylo Halytsky Lviv National Medical University. The samples were collected under the approval of the Bio-Ethics Review Board of the Danylo Halytsky Lviv National Medical University in accordance with the regulations of the Ministry of Health of Ukraine. Documented consent was obtained from all patients included in the study, and the informed consent form was also approved by the Bio-Ethics Review Board of the Danylo Halytsky Lviv National Medical University. Blood for antibody purification was obtained from two immunized mice. Animals were treated in compliance with the Council of Europe Convention on protection of vertebrate animals used for scientific purposes (approval by Bio-Ethics Review Board of the Institute of Cell Biology, NAS of Ukraine).

## Results and discussion

### Magnetic PHEMA microspheres

To easily detect autoimmune diseases such as MS in patient blood by affinity chromatography techniques, mgt.PHEMA microspheres conjugated with proteins are a very attractive approach. Therefore, starting monodisperse macroporous PHEMA particles, 4.3 µm in diameter, were developed employing multistep swelling polymerization of HEMA, MCMEMA, and EDMA according to the Ugelstad method, where inert solvents, such as CyAc and DBP, served as the porogen [30]. Subsequent hydrolysis of MCMEMA-containing PHEMA microspheres introduced COOH functionalities (∼1 mmol/g according to titration with NaOH). This procedure was followed by Fe(II) and Fe(III) chloride precipitation with ammonia inside the pores to yield iron oxides, which rendered the particles with magnetic properties enabling easy manipulation of the microspheres in a magnetic field [39]. Absence of particle aggregation in water and superior mechanical properties were additional advantages of these microspheres [40].

### Morphology, size, and composition of the microspheres

**Figure 1:**
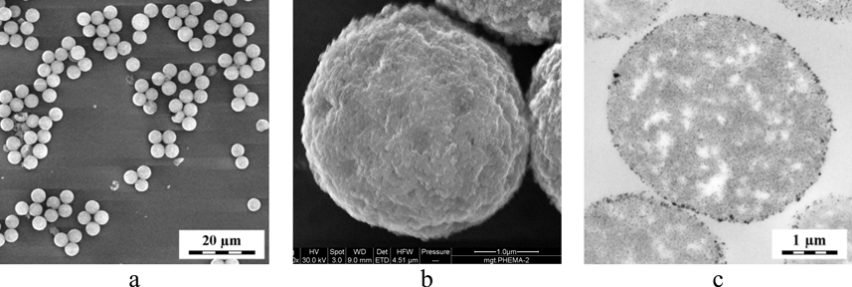
SEM micrographs of (a) neat PHEMA-COOH and (b) mgt.PHEMA microspheres. (c) TEM micrograph of a cross-section of mgt.PHEMA microspheres

Morphology and size of both neat PHEMA-COOH and mgt.PHEMA microspheres were documented by SEM micrographs (Figure 1a, b). All particles were monodispersed (PDI = 1.01), which is important for their future biomedical applications, where uniform physicochemical and biological properties are required. Mgt.PHEMA particles had a slightly smaller diameter (4.1 µm) than their non-magnetic counterparts (4.3 µm; Table 1), which can be explained by repetitive volume contractions and swelling during precipitation of iron oxides with ammonia and multiple washing with water. TEM micrograph of individual microsphere cross-sections confirmed fine and homogeneous distribution of iron oxides inside the pores (Figure 1c). Mgt.PHEMA microspheres were then easily separated in a magnetic field [39].

**Table 1:** Characterization of microspheres

Microspheres	*D*_n_^1^ (μm)	PDI^2^	C^3^ (wt.%)	H^3^ (wt.%)	N^3^ (wt.%)	Fe^4^ (wt.%)
PHEMA-COOH	4.3	1.01	50.4	7.1	–	–
Mgt.PHEMA	4.1	1.01	41.8	6	–	16.8

^1^Number-average particle size; ^2^polydispersity index; ^3,4^results of elemental analysis and AAS respectively.

To quantitatively describe porous characters of the PHEMA-COOH microspheres, their specific surface area (*S*_BET_), pore volume according to He adsorption (*V*_p_), cumulative (*V*_c_) and total (*V*_t_) pore volume, pore diameter (*d*), and porosity (*ε*) were determined. *S*_BET_ represents area of the microspheres accessible for nitrogen per unit mass, *V*_p_, *V*_c_, and *V*_t_ include pores accessible to helium, mercury, and cyclohexane or water respectively, and *ε* shows fraction of pores in the particles, the value of which depends on the detection method. According to the pore diameter, porous materials can be divided into micro- (*d* < 2 nm), meso- (2 < *d* < 50 nm), and macroporous (*d* > 50 nm) [41]. The presence of mesopores in the PHEMA-COOH microspheres was corroborated by rather low values of specific surface area (*S*_BET_ = 10 m^2^/g), pore size (*d* = 29 nm), pore volume (*V*_p_ = 0.07 ml/g), and porosity (*ε* = 9%), as determined by nitrogen and helium adsorption methods. This analysis was also in agreement with the mercury porosimetry results (*d* = 20 nm, *V*_c_ = 0.12 ml/g, and *ε* = 14%). To confirm macroporous character of the PHEMA-COOH microspheres, total pore volume *V*_t_ = WR or CXR was measured. CXR was rather small (0.5 ml/g), indicating low pore volume and porosity (*ε* = 39%) because cyclohexane does not swell the polymer. In contrast, PHEMA-COOH microspheres highly swelled in water (*V*_t_ = WR = 4 ml/g), indicating that porosity was high (*ε* = 84%). After subtracting the contribution of the mesopores from the total porosity, *ε* = 30% was ascribed to macropores and *ε* = 45% to PHEMA swelling.

**Figure 2:**
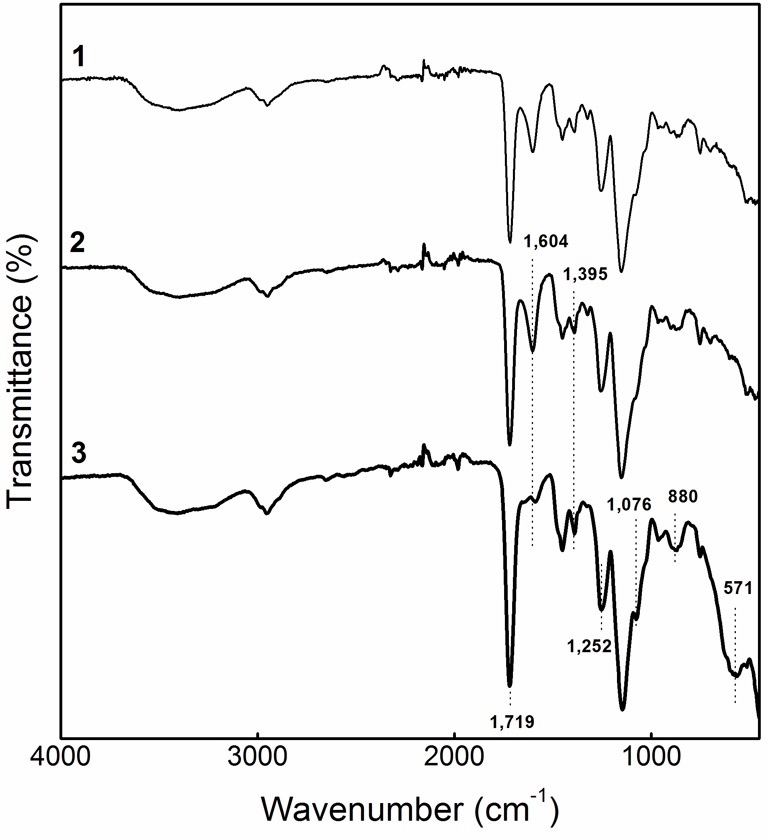
FTIR spectra of (1) neat PHEMA, (2) PHEMA-COOH, and (3) mgt.PHEMA microspheres

Comparison of the results from the elemental analysis of neat PHEMA-COOH and mgt.PHEMA microspheres revealed that C content decreased from 50 to 42 wt.%, while the Fe amount in the latter particles reached 17 wt.% (Table 1), corresponding to 24 wt.% of Fe_3_O_4_ or γ-Fe_2_O_3_. This amount of iron oxide is quite sufficient for good magnetic separation of the particles. The FTIR spectra of the neat PHEMA, PHEMA-COOH, and mgt.PHEMA microspheres are shown in Figure 2. The presence of carboxylate groups in PHEMA-COOH was documented by strong asymmetric and weak symmetric COO^−^ stretching vibrations at 1,604 and 1,395 cm^−1^ respectively. The former band disappeared in the spectrum of mgt.PHEMA due to acidification of particle suspension prior to iron oxide precipitation, confirming the introduction of COOH moieties. Moreover, carboxyl groups showed strong asymmetric C=O stretching and medium out-of-plane OH bending vibrations at 1,719 and 880 cm^−1^ respectively. Intense and weak bands at 1,252 and 1,076 cm^−1^ from C=O stretching involved interaction [42,43] with in-plane OH deformation at 1,395 cm^−1^. Spectra of non-magnetic and magnetic particles substantially differed in the presence of broad asymmetric Fe–O stretching vibrations at 571 cm^−1^, confirming γ-Fe_2_O_3_ formation inside the polymer matrix [44]. It should be noted that some bands in the mgt.PHEMA spectrum overlapped due to an Fe–O-induced shielding effect [45,46].

### Antibody purification with p46/Myo1C-mgt.PHEMA microspheres

**Figure 3:**
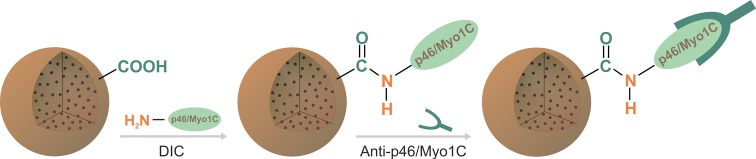
Binding of p46/Myo1C antigen on mgt.PHEMA particle and capture of monospecific anti-p46/Myo1C antibody

**Figure 4:**
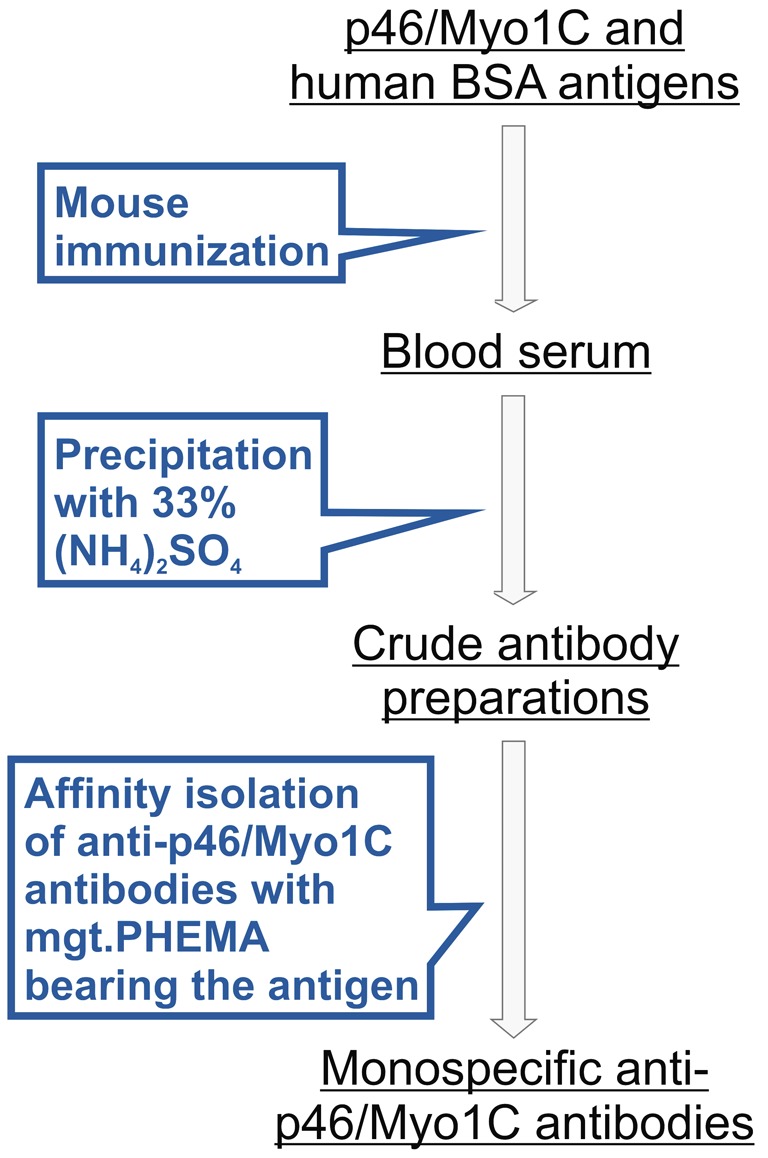
Schematic view of monospecific anti-p46/Myo1C antibody isolation by antigen-containing p46/Myo1C-mgt.PHEMA microspheres

Protein p46/Myo1C from blood serum serves as a potential molecular marker of selected autoimmune diseases, particularly MS [47]. Determination of anti-p46/Myo1C antibodies in blood of autoimmune patients is thus very important for diagnostics, prediction of disease development, and effectiveness of treatment. For this purpose, p46/Myo1C antigen was bound on the mgt.PHEMA microspheres via DIC activation, and the monospecific antibody was captured (Figure 3). Isolation of anti-p46/Myo1C by antigen-containing p46/Myo1C-mgt.PHEMA microspheres is schematically presented in Figure 4. The first step includes mouse immunization with crude preparation of TCA-extracted proteins from MS patient blood serum (p46/Myo1C) and human blood serum albumin as a contaminant. This step is followed by precipitation of the anti-p46/Myo1C antibodies with 33% (NH_4_)_2_SO_4_ from crude IgG preparation present in the blood serum of the immunized animals and further one-step affinity purification of antibodies with p46/Myo1C-mgt.PHEMA microspheres (Figure 4).

**Figure 5:**
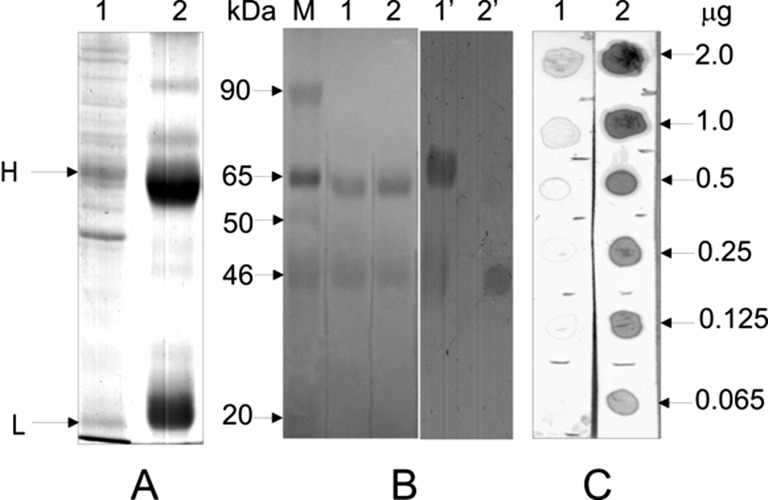
Characterization of anti-human p46/Myo1C antibodies after affinity purification from blood serum of immunized mice by p46/Myo1C-mgt.PHEMA microspheres (**A**) SDS/PAGE (10% polyacrylamide) electrophoregram of proteins precipitated with 33% (NH_4_)_2_SO_4_ (lane 1; 10 µg of protein) and after affinity purification (lane 2; 15 µg of protein). H and L—heavy and light chains of IgGs respectively. (**B**) Western blot analysis of TCA-extracted proteins. M—protein standards; 1, 2—Ponceau S-stained membrane; 1’, 2’—membrane strips treated with (NH_4_)_2_SO_4_ and affinity purified antibodies. (**C**) Dot blot analysis of TCA-extracted proteins precipitated with (NH_4_)_2_SO_4_ (lane 1) and affinity purified mouse antibodies (lane 2). Arrows on the right side show the amount of p46/Myo1C loaded on the nitrocellulose membrane.

High efficiency of the affinity purification is demonstrated in Figure 5, which shows the results of affinity-purified anti-human p46/Myo1C antibody characterization by SDS/PAGE, Western blot, and dot blot analyses. Highly purified antibodies were obtained without contamination by the antibodies against BSA (Figure 5A, C, lane 2 and Figure 5B, lane 2’). Dot blot analysis confirmed high titer of these specific antibodies, demonstrating efficient immune reactivity in doses as low as 0.065 µg of protein (Figure 5C).

## Conclusions

MS is the most common chronic inflammatory disease of the central nervous system (CNS) with supposed autoimmune etiology. MS deaths increased in the period of 1990–2013 from 12 to 20 thousand people per year [48]; therefore, many diagnostic and therapeutic techniques are now under development. However, no reliable method of immune processes characterization exists. Detection of biomarkers in body fluids may thus facilitate diagnosis and prediction of disease progression [49]. New analytical methods often investigate anti-myelin antibodies, inducing CNS demyelination, which are obtained from blood serum and cerebrospinal fluid. In clinical practice, serum is preferable due to its simple availability from the patient’s body [50–52]. Recently, unconventional human myosin p46/Myo1C was detected as a potential MS marker [26]; however, there was need of preconcentration and rapid quantification of monospecific antibodies against this protein. To solve this problem, affinity chromatography on magnetic polymer particles with attached specific protein antigens was suggested. In our procedure, mgt.PHEMA microspheres were selected due to their blood compatibility, non-toxicity, and widespread biomedical applications [53,54]. However, PHEMA hydrophobically interacts with some proteins, necessitating modification of the particles, e.g. with polysaccharides, to immobilize affinity ligands [55,56]. Magnetic polymer microspheres are commonly prepared by the suspension polymerization in the presence of a magnetic fluid [57], which unfortunately produces particles of a broad size distribution and low saturation magnetization. For this reason, more sophisticated Ugelstad’s multistep swelling polymerization ensuring formation of porous microspheres uniform in size and of identical physical, chemical, and biological properties was employed in this work. After creating iron oxides inside the PHEMA particles, they were easily separable in the magnetic field. These new microspheres with immobilized p46/Myo1C protein isolated from blood serum of MS patients were then found to be effective for affinity purification of the monospecific anti-p46/Myo1C antibodies, reaching a detection limit as low as 0.065 µg of protein. In general, the newly developed mgt.PHEMA microspheres can be conjugated with any specific antigen present in cells of patients suffering from neurological disorders and can be exploited for highly sensitive affinity isolation of biomarkers. This approach may facilitate both diagnosis and treatment of autoimmune diseases.

## Abbreviations

AAS: atomic absorption spectroscopy
ABTB: 2,2′-azobis(2,3,3-trimethylbutanonitrile)
CNS: central nervous system
CXR: cyclohexane regain
CyAc: cyclohexyl acetate
DAB: 3,3′-diaminobenzidine
DBP: dibutyl phthalate
DIC: *N,N*′-diisopropylcarbodiimide
EDMA: ethylene dimethacrylate
FTIR: Fourier transform infrared
HEMA: 2-hydroxyethyl methacrylate
MCMEMA: 2-[(methoxycarbonyl)methoxy]ethyl methacrylate
mgt.PHEMA: magnetic poly(2-hydroxyethyl methacrylate)
MS: multiple sclerosis
PDI: polydispersity index
PHEMA: poly(2-hydroxyethyl methacrylate)
RA: rheumatoid arthritis
SLE: systemic lupus erythematosus
TCA: trichloroacetic acid
WR: water regain

## References

[1] Setchell C.H. (1985) Magnetic separations in biotechnology: a review. J. Chem. Technol. Biotechnol. 35, 175–182

[2] Taguchi T., Arakaki A., Takeyama H., Haraguchi S., Yoshino M., Kaneko M. (2007) Detection of *Cryptosporidium parvum* oocysts using a microfluidic device equipped with the SUS micromesh and FITC-labeled antibody. Biotechnol. Bioeng. 96, 272–280 16917954 10.1002/bit.21104

[3] Tartaj P., Morales M.D., Veintemillas-Verdaguer S., González-Carreño T. and Serna C.J. (2003) The preparation of magnetic nanoparticles for applications in biomedicine. J. Phys. D-Appl. Phys. 36, R182–R197

[4] Mallakpour S. and Madani M. (2015) A review of current coupling agents for modification of metal oxide nanoparticles. Prog. Org. Coat. 86, 194–207

[5] Bautista M.C., Bomati-Miguel O., del Puerto Morales M., Serna C.J. and Veintemillas-Verdaguer S. (2005) Surface characterization of dextran-coated iron oxide nanoparticles prepared by laser pyrolysis and coprecipitation. J. Magn. Magn. Mater. 293, 20–27

[6] Basti H., Tahar L.B., Smiri L.S., Herbst F., Nowak S., Mangeney C. (2016) Surface modification of γ-Fe_2_O_3_ nanoparticles by grafting from poly-(hydroxyethylmethacrylate) and poly-(methacrylic acid): qualitative and quantitative analysis of the polymeric coating. Colloid Surf. A 490, 222–231

[7] Wang W.-C., Neoh K.-G. and Kang E.-T. (2006) Surface functionalization of Fe_3_O_4_ magnetic nanoparticles via RAFT-mediated graft polymerization. Macromol. Rapid Commun. 27, 1665–1669

[8] Wan S.R., Huang J.S., Yan H.S. and Liu K.L. (2006) Size-controlled preparation of magnetite nanoparticles in the presence of graft copolymers. J. Mater. Chem. 16, 298–303

[9] Ngaboni-Okassa L., Marchais H., Douziech-Eyrolles L., Cohen-Jonathan S., Soucé M., Dubois P. (2005) Development and characterization of sub-micron poly(D,L-lactide-*co*-glycolide) particles loaded with magnetite/maghemite nanoparticles. Int. J. Pharm. 302, 187–196 16099119 10.1016/j.ijpharm.2005.06.024

[10] Deng Y., Wang L., Yang W., Fu S. and Elaïssari A. (2003) Preparation of magnetic polymeric particles via inverse microemulsion polymerization process. J. Magn. Magn. Mater. 257, 69–78

[11] Ramirez L.P. and Landfester K. (2003) Magnetic polystyrene nanoparticles with a high magnetite content obtained by miniemulsion processes. Macromol. Chem. Phys. 204, 22–31

[12] Jin H., Lin J.M., Wang X., Xin T.B., Liang S.X., Li Z.J. (2009) Magnetic particle-based chemiluminescence enzyme immunoassay for free thyroxine in human serum. J. Pharm. Biomed. Anal. 50, 891–896 19581068 10.1016/j.jpba.2009.06.011

[13] Zhang R.Q., Nakajima H., Soh N., Nakano K., Masadome T., Nagata K. (2007) Sequential injection chemiluminescence immunoassay for nonionic surfactants by using magnetic microbeads. Anal. Chim. Acta 600, 105–113 17903471 10.1016/j.aca.2007.02.052

[14] Zhang Q.Y., Chen H., Lin Z. and Lin J.-M. (2012) Comparison of chemiluminescence enzyme immunoassay based on magnetic microparticles with traditional colorimetric ELISA for the detection of serum α-fetoprotein. J. Pharm. Anal. 2, 130–135 10.1016/j.jpha.2011.10.001 PMC5760820 29403732

[15] Fang F.F., Kim J.H. and Choi H.J. (2009) Synthesis of core-shell structured PS/Fe_3_O_4_ microbeads and their magnetorheology. Polymer 50, 2290–2293

[16] Arica M.Y., Yavuz H., Patir S. and Denizli A. (2000) Immobilization of glucoamylase onto spacer-arm attached magnetic poly(methylmethacrylate) microspheres: Characterization and application to a continuous flow reactor. J. Mol. Catal. B-Enzym. 11, 127–138

[17] Kuan W.-C., Horák D., Plichta Z. and Lee W.-C. (2014) Immunocapture of CD133-positive cells from human cancer cell lines by using monodisperse magnetic poly(glycidyl methacrylate) microspheres containing amino groups. Mater. Sci. Eng. C 34, 193–200 10.1016/j.msec.2013.09.009 24268249

[18] Taitt C.R., Shriver-Lake L.C., Anderson G.P. and Ligler F.S. (2011) Surface modification and biomolecule immobilization on polymer spheres for biosensing applications. Methods Mol. Biol. 726, 77–94 21424444 10.1007/978-1-61779-052-2_6

[19] Horák D. (1992) The use of poly(2-hydroxyethyl methacrylate) in medicine. Chem. Listy 86, 681–691

[20] Horák D., Červinka M. and Půža V. (1997) Hydrogels in endovascular embolization VI. Toxicity tests of poly(2-hydroxyethyl methacrylate) particles on cell cultures. Biomaterials 18, 1355–1359 9363335 10.1016/s0142-9612(97)00059-8

[21] Yavuz H., Ozden K., Kin E.P. and Denizli A. (2009) Concanavalin A binding on PHEMA beads and their interactions with myeloma cells. J. Macromol. Sci. Part A-Pure Appl. Chem. 46, 163–169

[22] Chouhan R. and Bajpai A.K. (2010) Release dynamics of ciprofloxacin from swellable nanocarriers of poly(2-hydroxyethyl methacrylate): An in vitro study. Nanomed.-Nanotechnol. Biol. Med. 6, 453–462 10.1016/j.nano.2009.11.006 20044034

[23] Adkins J.N., Varnum S.M., Auberry K.J., Moore R.J., Angell N.H., Smith R.D. (2002) Toward a human blood serum proteome: analysis by multidimensional separation coupled with mass spectrometry. Mol. Cell. Proteomics 1, 947–955 12543931 10.1074/mcp.m200066-mcp200

[24] Schrader M. and Schulz-Knappe P. (2001) Peptidomics technologies for human body fluids. Trends Biotechnol. 19, S55–S60 11780972 10.1016/S0167-7799(01)01800-5

[25] Wrotnowski C. (1998) The future of plasma proteins. Genet. Eng. News 18, 14–18

[26] Myronovkij S., Negrych N., Nehrych T., Redowicz M.J., Souchelnytskyi S., Stoika R. (2016) Identification of a 48 kDa form of unconventional myosin 1c in blood serum of patients with autoimmune diseases. Biochem. Biophys. Rep. 5, 175–179 10.1016/j.bbrep.2015.12.001 PMC5600340 28955821

[27] Horák D., Plichta Z., Starykovych M., Myronovskij S., Kit Y., Chopyak V. (2015) Calf thymus histone-conjugated magnetic poly(2-oxoethyl methacrylate) microspheres for affinity isolation of anti-histone IgGs from the blood serum of patients with systemic lupus erythematosus. RSC Adv. 5, 63050–63055

[28] Kubinová Š., Horák D. and Syková E. (2009) Cholesterol-modified superporous poly(2-hydroxyethyl methacrylate) scaffolds for tissue engineering. Biomaterials 30, 4601–4609 19500833 10.1016/j.biomaterials.2009.05.007

[29] Overberger C.G., Oshaughnessy M.T. and Shalit H. (1949) The preparation of some aliphatic azo nitriles and their decomposition in solution. J. Am. Chem. Soc. 71, 2661–2666

[30] Ugelstad J., Ellingsen T., Berge A. and Helgee B. (1983) Magnetic polymer particles and process for the preparation thereof. Eur. Pat. WO 83/03920

[31] Horák D., Kučerová J., Korecká L., Jankovičová B., Palarčík J., Mikulášek P. (2012) New monodisperse magnetic polymer microspheres biofunctionalized for enzyme catalysis and bioaffinity separations. Macromol. Biosci. 12, 647–655 22411761 10.1002/mabi.201100393

[32] Horák D., Hlídková H., Hiraoui M., Taverna M., Proks V., Mázl Chánová E. (2014) Monodisperse carboxyl-functionalized poly(ethylene glycol)-coated magnetic poly(glycidyl methacrylate) microspheres: Application to the immunocapture of β-amyloid peptides. Macromol. Biosci. 14, 1590–1599 25142028 10.1002/mabi.201400249

[33] Marhol M. (1976) Ion Exchangers in Chemistry and Radiochemistry, pp. 75, Academia, Prague

[35] Rigby S.P., Barwick D., Fletcher R.S. and Riley S.N. (2003) Interpreting mercury porosimetry data for catalyst supports using semi-empirical alternatives to the Washburn equation. Appl. Catal. A 238, 303–318

[36] Horák D., Kroupová J., Šlouf M. and Dvořák P. (2004) Poly(2-hydroxyethyl methacrylate)-based slabs as a mouse embryonic stem cell support. Biomaterials 25, 5249–5260 15110476 10.1016/j.biomaterials.2003.12.031

[37] Štamberg J. and Ševčík S. (1966) Chemical transformations of polymers III. Selective hydrolysis of a copolymer of diethylene glycol methacrylate and diethylene glycol dimethacrylate. Collect. Czech. Chem. Commun. 31, 1009–1016

[38] Kit Y., Bilyy R., Stoika R., Mitina N. and Zaichenko A. (2010) Immunogenicity and adjuvant properties of novel biocompatible nanoparticles. In Biocompatible Nanomaterials: Synthesis, Characterization and Applications (Kumar S.A., Thiagarajan S. and Wang S.-F., eds), pp. 209–223, Nova Sci. Publ., Hauppauge, New York

[39] Horák D., Svobodová Z., Autebert J., Coudert B., Plichta Z., Královec K. (2013) Albumin-coated monodisperse magnetic poly(glycidyl methacrylate) microspheres with immobilized antibodies: Application to the capture of epithelial cancer cells. J. Biomed. Mater. Res. Part A 101, 23–32 10.1002/jbm.a.34297 22767416

[40] Horák D., Švec F., Ilavský M., Bleha M., Baldrián J. and Kálal J. (1981) Reactive polymers XXXVI. The effect of polymerization conditions on the porosity and mechanical properties of macroporous suspension copolymers from glycidyl methacrylate-ethylene dimethacrylate. Angew. Makromol. Chem. 95, 117–127

[41] Rouquerol J., Avnir D., Fairbridge C.W., Everett D.H., Haynes J.M., Pernicone N. (1994) Recommendations for the characterization of porous solids. Pure Appl. Chem. 66, 1739–1758

[42] Colthup N.B., Daly L.H. and Wiberley S.E. (1990) Introduction to Infrared and Raman Spectroscopy, 3rd edn, Academic Press, San Diego

[43] Silverstein R.M., Bassler G.C. and Morrill T.C. (1991) Spectrometric Identification of Organic Compounds, 5th edn, Wiley, New York

[44] Sidhu P.S. (1988) Transformation of trace element-substituted maghemite to hematite. Clay Clay Min 36, 31–38

[45] Mou X., Li Y., Zhang B., Yao L., Wei X., Su D.S. (2012) Crystal-phase- and morphology-controlled synthesis of Fe_2_O_3_ nanomaterials. Eur. J. Inorg. Chem. 2012, 2684–2690

[46] Chen D. and Xu R. (1998) Hydrothermal synthesis and characterization of nanocrystalline γ-Fe_2_O_3_ particles. J. Solid State Chem. 137, 185–190

[47] Mollenhauer B., Esselmann H., Roeber S., Schulz-Schaeffer W.J., Trenkwalder C., Bibl M. (2011) Different CSF β-amyloid processing in Alzheimer's and Creutzfeldt-Jakob disease. J. Neural Transm. 118, 691–697 21210287 10.1007/s00702-010-0543-z

[48] GBD 2013 Mortality and Causes of Death Collaborators (2015) Global, regional, and national age-sex specific all-cause and cause-specific mortality for 240 causes of death, 1990-2013: A systematic analysis for the Global Burden of Disease Study 2013. Lancet 385, 117–171 25530442 10.1016/S0140-6736(14)61682-2 PMC4340604

[49] Miller A.E. (2011) Multiple sclerosis: where will we be in 2020. Mt. Sinai J. Med. 78, 268–279 21425270 10.1002/msj.20242

[50] D’Ambrosio A., Pontecorvo S., Colasanti T., Zamboni S., Francia A. and Margutti P. (2015) Peripheral blood biomarkers in multiple sclerosis. Autoimmun. Rev. 14, 1097–1110 26226413 10.1016/j.autrev.2015.07.014

[51] Akgöl S., Özkara S., Uzun L., Yılmaz F. and Denizli A. (2007) Pseudospecific magnetic affinity beads for immunoglobulin-g depletion from human serum. J. Appl. Polym. Sci. 106, 2405–2412

[52] Özkara S., Akgöl S., Çanak Y. and Denizli A. (2004) A novel magnetic adsorbent for immunoglobulin-G purification in a magnetically stabilized fluidized bed. Biotechnol. Prog. 20, 1169–1175 15296444 10.1021/bp049896s

[53] Denizli A., Rad A.Y. and Pişkin E. (1995) Protein A immobilized polyhydroxyethylmethacrylate beads for affinity sorption of human immunoglobulin G. J. Chromatogr. B 668, 13–19 10.1016/0378-4347(95)00047-m 7550969

[54] Rittich B., Španová A. and Horák D. (2009) Functionalized magnetic microspheres with hydrophilic properties for molecular diagnostic applications. Food Res. Int. 42, 493–498

[55] Deyl Z. and Mikšík I. (2000) Advanced separation methods for collagen parent α-chains, their polymers and fragments. J. Chromatogr. B 739, 3–31 10.1016/s0378-4347(99)00515-0 10744310

[56] Štulík K., Pacáková V., Suchánková J. and Claessens H.A. (1997) Stationary phases for peptide analysis by high performance liquid chromatography: A review. Anal. Chim. Acta 352, 1–19

[57] Liu X., Guan Y., Shen R. and Liu H. (2005) Immobilization of lipase onto micron-size magnetic beads. J. Chromatogr. B 822, 91–97 10.1016/j.jchromb.2005.06.001 15998604

